# Perigastric abscess caused by delayed perforation after gastric endoscopic submucosal dissection: successful conservative treatment without perforation closure: a case report

**DOI:** 10.1186/s13256-023-03785-5

**Published:** 2023-03-14

**Authors:** Shinya Nagae, Yoshiaki Kimoto, Rikimaru Sawada, Koichi Furuta, Yohei Ito, Nao Takeuchi, Syunya Takayanagi, Yuki Kano, Rindo Ishii, Takashi Sakuno, Ryoju Negishi, Kohei Ono, Yohei Minato, Takashi Muramoto, Ken Ohata

**Affiliations:** Department of Gastrointestinal Endoscopy, NTT Medical Center Tokyo, 5-9-22 Higashi-Gotanda, Shinagawa-Ku, Tokyo, 141-8625 Japan

**Keywords:** Endoscopic submucosal dissection, Delayed perforation, Intraperitoneal abscess, Conservative treatment, Adverse effects

## Abstract

**Background:**

Perigastric abscess caused by delayed perforation after endoscopic submucosal dissection is a very rare complication. In principle, delayed perforation after endoscopic submucosal dissection is treated surgically. Herein, we report a case of perigastric abscess caused by delayed perforation after gastric endoscopic submucosal dissection that was treated conservatively, without perforation closure, and in which the patient was discharged from hospital in a short period.

**Case presentation:**

A-74-year-old Asian man was diagnosed with having early gastric cancer on follow-up endoscopy and was admitted to our hospital for endoscopic resection. Endoscopic submucosal dissection was performed without intraoperative complications. On postoperative day 2, the patient complained of a slight abdominal pain localized to the epigastric region and a small amount of melena. A computed tomography scan revealed the presence of free air in the peritoneal cavity, and a little fluid collection abutting the dorsal area of the stomach. An endoscopy examination showed a deep ulcer with the accumulation of pus, suggesting a perforation in the post-endoscopic submucosal dissection ulcer. We diagnosed a perigastric abscess, caused by delayed perforation after endoscopic submucosal dissection, and opted for conservative treatment, leaving the perforation site open to allow spontaneous drainage from the abscess into the stomach. A follow-up computed tomography scan revealed an encapsuled and localized perigastric abscess on postoperative day 5, and the disappearance of the free air and the regression of the perigastric abscess on postoperative day 7. A follow-up endoscopy examination on postoperative day 7 showed the closure of the perforation. Finally, surgery was avoided, and the patient was discharged on postoperative day 14, after a relatively short hospital stay.

**Conclusion:**

Regarding the treatment of perigastric abscess, caused by delayed perforation after endoscopic submucosal dissection, leaving the perforation site open to allow spontaneous drainage may shorten the conservative treatment period.

## Background

Perforation is a major complication of endoscopic submucosal dissection (ESD), as well as bleeding. The incidences of intraoperative and delayed perforation are about 2.3% and 0.4%, respectively [1]. Delayed perforation is rare but can cause peritonitis, requiring an emergency operation. Some reports, however, have concluded that, if peritonitis does not occur, conservative treatment with endoscopic closure of the perforation site is feasible [2, 3].

Although rare, delayed perforation after ESD can lead to a perigastric abscess. In some previous cases without peritonitis, abscesses were treated successfully in a conservative manner, but a long hospital stay was typically required [4–6].

In principle, delayed perforation after ESD is treated surgically [7]. Herein, we report a case of a perigastric abscess caused by delayed perforation after gastric ESD, which was treated conservatively without perforation closure, and in which the patient was discharged from hospital in a short period.

## Case presentation

**Fig. 1:**
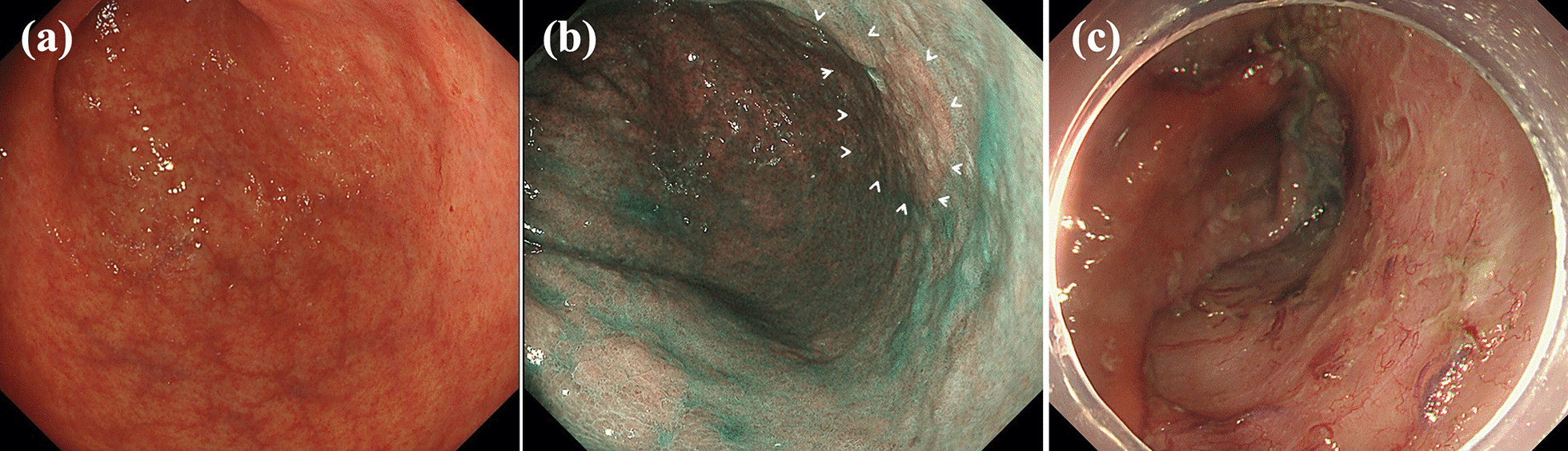
**a**, **b** An upper gastrointestinal endoscopy revealed an 18-mm faded, superficial flat lesion on the posterior wall of the gastric antrum. **c** No muscle injury, muscle layer exposure, and microperforation observed during endoscopic submucosal dissection

**Fig. 2:**
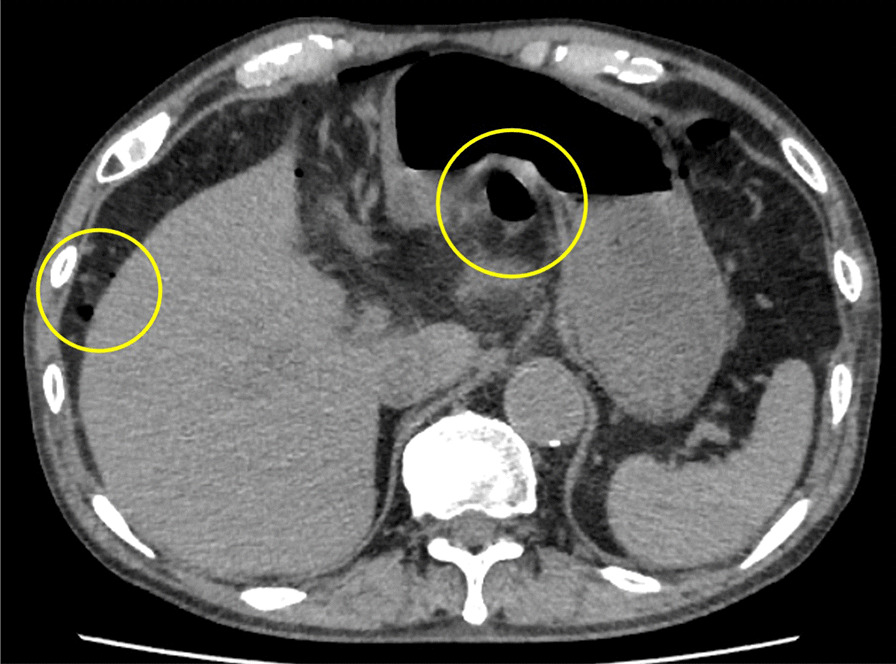
A computed tomography examination revealed the presence of free air in the peritoneal cavity, and a little fluid collection abutting the dorsal area of the stomach. Left circle in the figures shows the free air in the abdominal cavity and right circle shows the fluid collection caused by delayed perforation

**Fig. 3:**
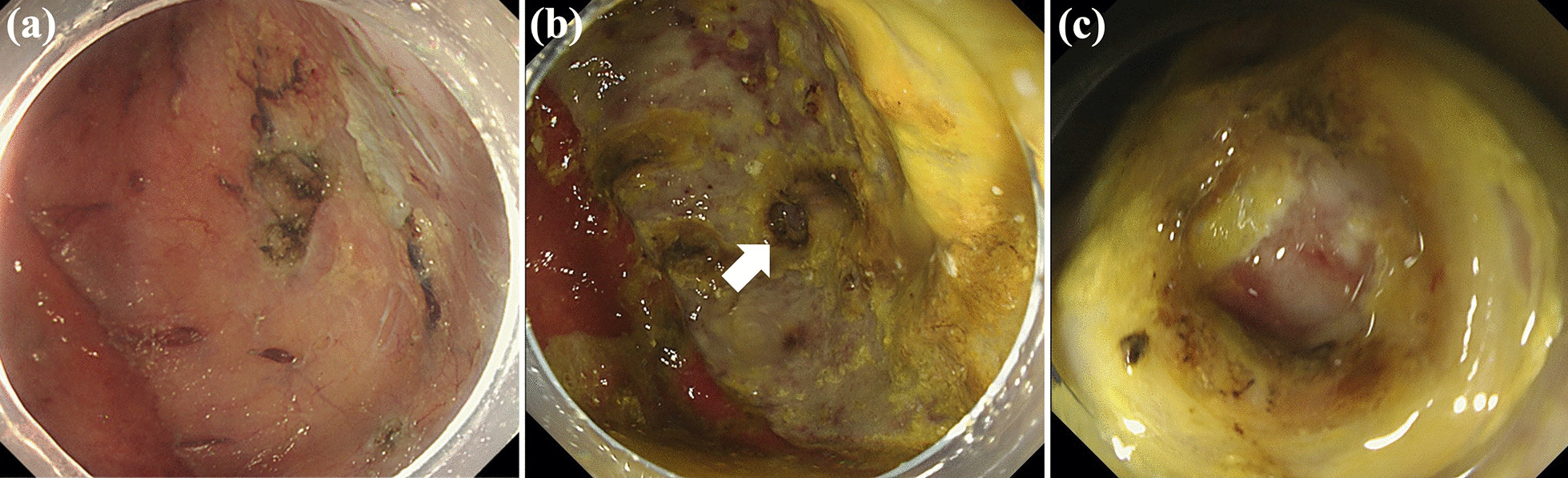
**a**–**c** Endoscopy on postoperative day 2 showed a deep ulcer, which had not been detected during endoscopic submucosal dissection, with accumulation of pus indicated the perforation in the post-endoscopic submucosal dissection ulcer. The arrow shows the site of perforation

**Fig. 4:**
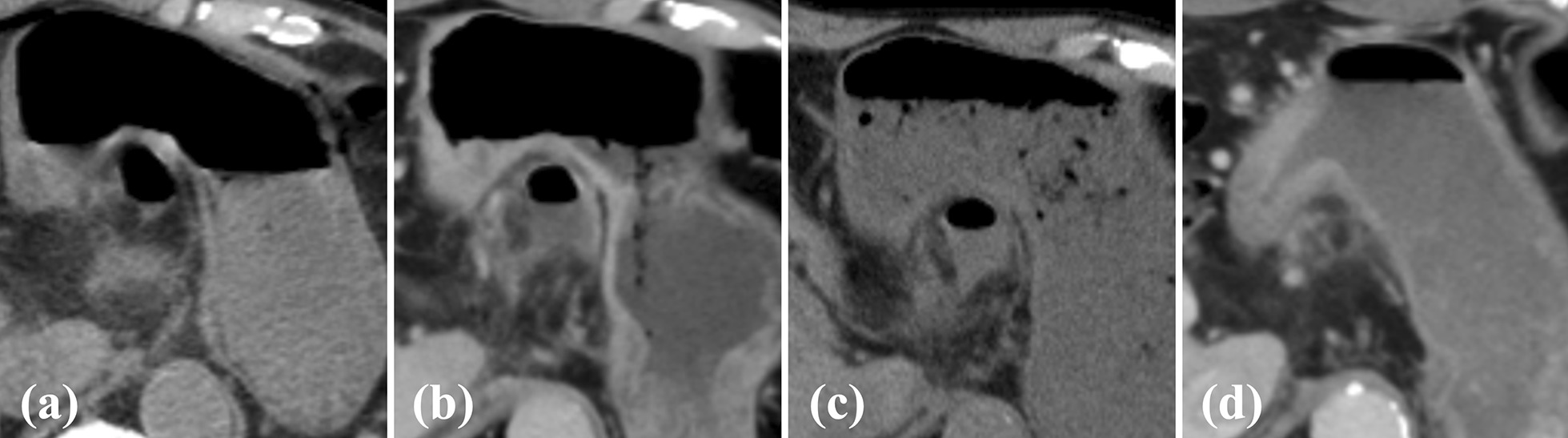
**a** On postoperative day 2, a computed tomography examination revealed the small amount of fluid collection abutting the dorsal area of the stomach. **b** On postoperative day 5, a follow-up computed tomography examination revealed an encapsuled and localized perigastric abscess. **c**, **d** The perigastric abscess had regressed on postoperative day 7 and had almost disappeared on postoperative day 34

A-74-year-old Asian man with a history of ESD for early gastric cancer (EGC) was found to have a second EGC during a follow-up endoscopy examination, and was admitted to our hospital for endoscopic resection. An upper gastrointestinal endoscopy examination revealed an 18 mm faded, superficial flat lesion on the posterior wall of the gastric antrum (Fig. 1a, b). A biopsy performed by a previous doctor resulted in the diagnosis of a signet ring cell carcinoma; therefore, we performed a negative biopsy to determine the excision range. ESD was performed using an insulated-tip (IT) knife 2 (KD-611 L; Olympus, Tokyo, Japan). The procedure took 1 hour and 40 minutes. The resected specimen measured 86 × 48 mm, which was histopathologically confirmed to be a signet ring cell carcinoma (28 × 25) limited to the lamina propria with no lymphovascular invasion. No intraoperative complications occurred (Fig. 1c), and the patient was asymptomatic during the operation. The patient developed a fever on postoperative day (POD) 1, but his general condition was stable. On POD2, the patient complained of a slight abdominal pain localized to the epigastric region and a small amount of melena. A computed tomography (CT) examination revealed the presence of free air in the peritoneal cavity, and a little fluid collection abutting the dorsal area of the stomach (Fig. 2). Since upper gastrointestinal bleeding was suspected, an endoscopy was performed on the same day, but no source of bleeding was found. Instead, a 5-mm, deep ulcer with the accumulation of pus was observed, suggesting a perforation of the post-ESD ulcer (Fig. 3b, c). We could detect the purulent pus flow from the abscess cavity to the stomach during endoscopy; therefore, we were convinced that the abscess communicated with the inside of the stomach. As the perforation site was lined with peritoneum, it was considered to be a gastric penetration. We diagnosed a perigastric abscess caused by delayed perforation after ESD. Blood tests revealed elevated C-reactive protein (CRP) levels (5.24 mg/dl, normal value < 0.3 mg/dl), but other than the patient’s body temperature (BT), his vital signs were stable (BT, 38.4 °C; respiratory rate, 18 breaths per minute; blood pressure, 129/81 mmHg; heart rate, 81 beats per minute), and there were no signs of peritonitis. After consultation with the surgical team, we opted for conservative treatment, which included fasting with intravenous fluid therapy, the administration of a proton pump inhibitor, and empirical antibiotics. We intentionally did not close the perforation and instead left it open to allow spontaneous drainage from the abscess into the stomach. Thereafter, the patient’s fever and abdominal pain improved gradually. CRP levels were elevated up to 12.38 mg/dl on POD5, but decreased to 5.34 mg/dl on POD7 and 1.67 mg/dl on POD14. A follow-up CT examination revealed an encapsuled and localized perigastric abscess on POD5 (Fig. 4b), and the disappearance of the free air and the regression of the perigastric abscess on POD7 (Fig. 4c). A follow-up endoscopy examination on POD7 showed the closure of the perforation. Oral feeding was resumed at this time. Finally, surgery was avoided, and the patient was discharged on POD14 after a relatively short hospital stay. Oral antibiotic treatment was continued for 2 weeks after hospital discharge, and a CT examination on POD34 showed no recurrence of the perigastric abscess (Fig. 4d).

## Discussion and conclusions

Delayed perforation after ESD is rare but can cause peritonitis, requiring surgery. If peritonitis does not occur, however, this complication can be treated conservatively. Ikezawa *et al.* reported a case that was treated conservatively with closure of a delayed perforation site using endoclips [2]. If the perforation site is small and can be closed promptly after perforation, the leakage of digestive juices into the peritoneal cavity can be minimized, allowing conservative treatment.

A few reports have described the formation of a perigastric abscess caused by delayed perforation after gastric ESD [4–6, 8, 9]. We previously reported a case of intraabdominal abscess caused by delayed perforation after ESD that was treated conservatively, with perforation closure and the EUS-guided insertion of a nasocavitary catheter drainage into the abscess [6]. Although the attachment of a polyglycolic acid sheet successfully closed the perforation, the patient’s clinical symptoms did not improve. Therefore, we inserted an additional drainage catheter into the abscess. Though this strategy allowed conservative treatment, the patient was not discharged from hospital until 40 days after ESD. The closure of the perforation site was thought to have enclosed the abscess cavity, delaying its spontaneous drainage and resulting in the prolonged hospital stay.

In this reported case, the perforation was not closed, and instead was left open to allow spontaneous drainage from the abscess into the stomach. This strategy might have contributed to the shorter hospital stay. Effective spontaneous drainage and the early resumption of oral feeding might promote the healing of the perforation and abscess.

We assumed that the following characteristics of perforations might determine the suitability of conservative treatment without perforation closure.

### Size

Large perforations have the potential to become chronic fistulas and are likely to be difficult to treat without closure of the fistula. Although a cutoff for perforation size is difficult to specify, the perforation in this reported case was small enough to close spontaneously without endoscopic closure.

### Encapsuled

If the abscess was not encapsuled, it would spread to other areas despite effective spontaneous drainage from the abscess into the stomach. In this reported case, we could detect that the abscess was encapsuled on POD5. Therefore, the abscess was localized and had regressed with conservative treatment.

Several previous reports have opted to close perforations during conservative treatment for delayed perforation after ESD [2, 3]. If an abscess has formed, however, leaving the perforation site open instead of closing it might be a better treatment option, and additional treatments (such as surgery or transgastric drainage) could be performed if the patient’s condition worsens.

Regarding the treatment of perigastric abscess caused by delayed perforation after ESD, leaving the perforation site open to allow spontaneous drainage may shorten the conservative treatment period.

## Data Availability

The datasets used and analyzed during the current study are available from the corresponding author on reasonable request.
